# Ex vivo gadoxetate relaxivities in rat liver tissue and blood at five magnetic field strengths from 1.41 to 7 T

**DOI:** 10.1002/nbm.4401

**Published:** 2020-08-26

**Authors:** Sabina Ziemian, Claudia Green, Steven Sourbron, Gregor Jost, Gunnar Schütz, Catherine D.G. Hines

**Affiliations:** ^1^ MR & CT Contrast Media Research Bayer AG Berlin Germany; ^2^ Department of Infection, Immunity and Cardiovascular Disease University of Sheffield Sheffield UK; ^3^ Translational Imaging Biomarkers Merck & Co, Inc. West Point Pennsylvania

**Keywords:** bile, blood, DCE‐MRI, gadoxetate, liver/hepatocytes, Mrp2‐KO rat, relaxivity, urine

## Abstract

Quantitative mapping of gadoxetate uptake and excretion rates in liver cells has shown potential to significantly improve the management of chronic liver disease and liver cancer. Unfortunately, technical and clinical validation of the technique is currently hampered by the lack of data on gadoxetate relaxivity. The aim of this study was to fill this gap by measuring gadoxetate relaxivity in liver tissue, which approximates hepatocytes, in blood, urine and bile at magnetic field strengths of 1.41, 1.5, 3, 4.7 and 7 T. Measurements were performed ex vivo in 44 female Mrp2 knockout rats and 30 female wild‐type rats who had received an intravenous bolus of either 10, 25 or 40 μmol/kg gadoxetate. T1 was measured at 37 ± 3°C on NMR instruments (1.41 and 3 T), small‐animal MRI (4.7 and 7 T) and clinical MRI (1.5 and 3 T). Gadolinium concentration was measured with optical emission spectrometry or mass spectrometry. The impact on measurements of gadoxetate rate constants was determined by generalizing pharmacokinetic models to tissues with different relaxivities. Relaxivity values (L mmol^−1^ s^−1^) showed the expected dependency on tissue/biofluid type and field strength, ranging from 15.0 ± 0.9 (1.41) to 6.0 ± 0.3 (7) T in liver tissue, from 7.5 ± 0.2 (1.41) to 6.2 ± 0.3 (7) T in blood, from 5.6 ± 0.1 (1.41) to 4.5 ± 0.1 (7) T in urine and from 5.6 ± 0.4 (1.41) to 4.3 ± 0.6 (7) T in bile. Failing to correct for the relaxivity difference between liver tissue and blood overestimates intracellular uptake rates by a factor of 2.0 at 1.41 T, 1.8 at 1.5 T, 1.5 at 3 T and 1.2 at 4.7 T. The relaxivity values derived in this study can be used retrospectively and prospectively to remove a well‐known bias in gadoxetate rate constants. This will promote the clinical translation of MR‐based liver function assessment by enabling direct validation against reference methods and a more effective translation between in vitro findings, animal models and patient studies.

## Graphical abstract

T1 relaxivity (r1) values of the liver‐specific MRI contrast agent gadoxetate were determined *ex vivo* on rat samples of liver, blood, urine and bile at five different magnetic field strengths up to 7 T. The r1 of gadoxetate in liver tissue was higher compared to blood; values notably decreased with increasing field strength to blood level at 7 T. Our findings are potentially substantive for functional liver analysis, utilizing gadoxetate‑enhanced MRI methods.

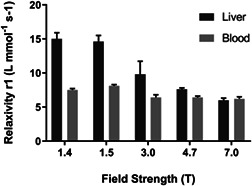

**Abbreviations used**
AALAC: Assessment and Accreditation of Laboratory Animal Care
ADME: absorption, distribution, metabolism and excretion
DCE‐MRI: dynamic contrast enhanced ‐ magnetic resonance imaging
EOB‐dtpa: ethoxybenzyl diethylenetriamine pentaacetic acid
GBCA: gadolinium‐based contrast agent
i.v.: intravenous
IACUC: Institutional Animal Care and Use Committee
ICP‐MS: inductively coupled plasma ‐ mass spectrometry
IR‐TSE: inversion recovery ‐ turbo spin‐echo
KO: knockout; p.i., postinjection
PBPK: physiologically based pharmacokinetic
R1: longitudinal relaxivity
R1: relaxation rate
RARE: rapid acquisition with relaxation enhancement
RD: recovery delay
ROI: region of interest
T1: longitudinal relaxation time
T1: estimated longitudinal relaxation time
TI: inversion time
TR: repetition time; wt, wild‐type

## 1 INTRODUCTION

Gadoxetate (Gd‐EOB‐DTPA) is a clinically well‐established, liver‐specific, gadolinium‐based contrast agent (GBCA)^1^, ^2^, ^3^ approved for the detection and characterization of focal liver lesions by magnetic resonance imaging (MRI).^4^ In healthy humans, ~50% of the administered dose is taken up by hepatocytes and is subsequently excreted into the bile, whereas the remaining 50% is excreted renally. In rats, ~70% of the dose is eliminated through the biliary route and ~30% renally.^5^, ^6^, ^7^

Specific uptake of gadoxetate by hepatocytes leads to T1‐shortening of liver parenchyma, while liver lesions show characteristic signal deviation from the parenchyma, thus contrasting the lesions. Genetic factors,^8^ chronic liver diseases^9^ and drug–drug interactions^10^, ^11^ may affect gadoxetate transport through hepatocytes, influencing liver parenchyma enhancement.

Gadoxetate kinetics in the liver can be displayed by spatially resolved dynamic gadoxetate‐enhanced liver MRI and may be utilized to evaluate hepatocyte function.^12^, ^13^, ^14^, ^15^ The quantification of liver function in vivo by dynamic gadoxetate‐enhanced MRI depends on determination of the gadoxetate concentrations in the relevant liver compartments, foremost in blood and hepatocytes, and potentially also in bile. Deriving concentrations from MRI signal intensities (SI) requires knowledge of the gadoxetate relaxivity (r1). Relaxivity is defined as the change in relaxation rate (R1) per unit concentration, and characterizes the efficacy of a GBCA for MR signal enhancement.^16^ GBCA r1 depends on factors like temperature, interaction with the chemical environment and magnetic field strength. With known values for r1 for a given environment, the contrast agent's concentration can be calculated from the determined relaxation rates in an MRI experiment.^17^

Gadoxetate r1 has been reported in the literature for water, blood, plasma, bile and for liver only at selected magnetic field strengths.^6^, ^18^, ^19^, ^20^ One report estimated gadoxetate r1 in liver tissue from wild‐type (wt) rats at 0.47 T to be a factor of 1.5 higher compared with blood,^6^ whereas another report presented a slightly higher relaxivity in liver compared with other tissues or fluids from wt rats at 1.5 T.^18^ Both studies analyzed liver tissue and not hepatocytes. This is considered to be highly relevant, as liver tissue contains additional compartments that can confound gadoxetate isolation during tissue collection. Biliary gadoxetate could greatly impact the results due to its high concentration. In addition, to our knowledge no literature data for liver gadoxetate r1 are available at field strengths higher than 1.5 T.

Given that no in vivo imaging occurs at 0.47 T, and that models use 0.47 T relaxivity values for tissue and blood, errors in gadoxetate concentration calculations may exist due to these assumptions. Further, relaxivity values reported for liver tissue do not reflect hepatocytes, which have different functional roles with gadoxetate than the liver in its entirety. Additionally, the relationship of relaxivity values between different tissue compartments cannot be assumed to scale linearly with field strength, due to other factors, such as viscosity and protein content. Relaxivity values in hepatocytes are needed to quantify rate constants, but values are not known and rate constants in the literature are inaccurate and not comparable between field strengths. The aim of this paper is to resolve this problem by providing reliable measurements of relaxivity at all relevant magnetic field strengths for MRI examinations. Therefore, we determined r1 numerical values for gadoxetate in liver tissue aiming at hepatocytes, in blood, urine and bile of rats ex vivo at 1.41, 1.5, 3, 4.7 and 7 T. ex vivo measurements are the only setting in which both relaxation rates and Gd concentration can be determined at the same time point in one experiment. Extracted tissue collected from the same animal reflects physiological levels of gadoxetate and its absorption, distribution, metabolism and excretion (ADME). Our findings will have implications for quantitative gadoxetate‐enhanced liver imaging. In particular, the relaxivity values for liver tissue and blood will impact on the kinetic modeling of dynamic gadoxetate‐enhanced MRI in the liver for quantitative estimation of liver excretory function and functional heterogeneity.

## 2 EXPERIMENTAL

### 2.1 Tissue sampling

Studies were performed at two different sites, hereafter referred to as “site 1” and “site 2”, and on two different rat strains. All animal protocols were compliant with and approved by the respective animal ethics regulations (German Animal Protection Law or IACUC guidelines at the AALAC‐accredited facilities).

Rats were housed in micro‐isolator cages, and housing rooms were maintained with controlled humidity and temperature (22°C) at 12‐hour light–dark cycles. All animals were provided nestlets and ad libitum access to standard chow and water. The physical condition of the animals was continuously monitored.

For ex vivo relaxivity measurements, in total, 44 female SD‐*Abcc2* ^*tm1sage*^ rats (Mrp2‐KO; Horizon Discovery Group, Cambridge, UK, and St. Louis, MO, USA), 29 rats at site 1 and 15 rats at site 2, with a weight range of 240‐310 g at the time of the experiment; and 30 female wt rats (WI:Crl [Han], Charles River, Sulzfeld, Germany, and MA, USA), 15 wt rats at site 1 and 15 wt rats at site 2, with a weight range of 190‐270 g, were used.

MRI SI, NMR SI and tissue gadolinium concentrations were determined in liver tissue from all wt and Mrp2‐KO animals, as well as in blood and in urine from all Mrp2‐KO rats. Bile gadolinium concentrations were further determined from all wt rats and 15 Mrp2‐KO animals at site 1. Anesthesia was initiated with a ketamine (Ketaset, Zoetis Deutschland GmbH, Berlin, Germany) and xylazine mixture (Rompun 2%, Bayer Vital GmbH, Leverkusen, Germany) at a ratio of 1 + 2, 1 mL/kg i.p. and maintained intravenously as continuous tail vein infusion at 3.5 mL/hour with the mixture diluted with saline 1:50 at site 1. At site 2, anesthesia was induced and sustained using a mixture of 2% isoflurane (Baxter Healthcare Corporation, Deerfield, IL, USA) in medical grade air. During the entire experiment, body temperature was maintained with a heating pad or warm water jacket and the animals were constantly monitored for respiratory rate and body temperature.

All the animals were then prepared for fluid collection. To enable blood collection in Mrp2‐KO rats, a catheter was placed in the left carotid artery (site 1) or in the femoral artery (site 2). Further, a cannula was inserted directly into the bladder with a PE50 tube for urine collection from Mrp2‐KO rats after occlusion of the urethra exit (site 1) or by direct insertion of a needle connected to a syringe into the bladder (site 2). To enable bile collection in both rat strains, the bile duct was cannulated by surgical implantation of 100 mm PE50 tubing into the bile duct at both sites.

After preparation for fluid collection, all the rats were randomly divided into three groups of different gadoxetate doses. Each group of rats received an i.v. bolus of either 10, 25 or 40 μmol/kg disodium gadoxetate (Primovist, Bayer Vital GmbH, Leverkusen, Germany or Eovist, Bayer HealthCare Pharmaceuticals Inc., Whippany, NJ, USA) into the tail vein, followed by a 500 μL saline flush. Blood was sampled at specific time points of 1 and 30 minutes postgadoxetate injection in heparinized tubes, whereas urine and bile were collected continuously for 30 minutes postinjection (p.i.). Animals were euthanized by exsanguination (site 1) or inhalation of 10%‐30% CO_2_ in air (site 2) 30 minutes after GBCA injection. Immediately after sacrificing the animals, wt rat and Mrp2‐KO rat liver tissue was dissected by taking six samples from different positions in the liver, avoiding inclusion of visible vessels and collecting ducts. Samples were placed into NMR (site 1) or Eppendorf (site 2) tubes, avoiding compression or entrapped air for NMR (n = 3) and MRI (n = 3) measurements. Aliquots of blood, urine and bile were placed into NMR tubes at a volume of 200 μL for NMR measurements and at a volume of 400 μL into NMR (site 1) or Eppendorf (site 2) tubes for MRI measurements.

All samples were kept at 37°C until measurement using a water bath or a heating pad throughout the course of tissue collection then transferred to the MRI scanner in tempered foam holders.

### 2.2 Relaxation measurements

Relaxation measurements of ex vivo samples were conducted separately after each animal sacrifice and were started directly after tissue or biofluid sample collection. All measurements were performed at 37 ± 3°C and the temperature was controlled throughout all measurements. In this study, T1 measurements were conducted on two NMR instruments and four MRI scanners, two dedicated small‐animal scanners and two clinical scanners. NMR and MRI sequence parameters are provided in Table 1.

**TABLE 1:** Imaging and spectroscopy parameters for T1 relaxation measurements

Coil	1.41 T	1.5 T	3 T	3 T	4.7 T	7 T
	dedicated probe	Knee		dedicated probe	72 id single channel volume	
**Sequence**	IR	IR‐TSE		IR	Variable TR TSE (RARE)	
**Turbo factor**	‐	25	20	‐	8	
**TE/echo spacing (ms)**	‐	8.4	9.5	‐	7.5/30	
**TI (ms)**	0.1 x T1e – 4 x T1e	25, 50, 100, 150, 200, 300, 500, 700, 1100, 1600, 2400, 4000		0.1 x T1e – 4 x T1e	‐	
**RD/TR (ms)**	5 x T1e	4975, 4950, 4900, 4800, 4700, 4500, 4300, 3900, 3400, 2600,1000		5 x T1e	100, 200, 300, 500, 750, 1200, 2000, 5500	
**Resolution (mm)**	‐	0.4 × 0.4 × 3		‐	0.45 × 0.45 × 1.16	

Abbreviations: id, internal diameter; IR‐TSE, inversion recovery ‐ turbo spin‐echo; RARE, Rapid Acquisition with Relaxation Enhancement; RD, recovery delay; T1e, estimated T1 relaxation time; TE, echo time; TI, inversion time, TR, repetition time.

We employed the following field strengths: 1.41 T (Minispec mq60, Bruker Analytik, Karlsruhe, Germany), 1.5 T (MRT; Avanto, Siemens Healthcare GmbH, Erlangen, Germany), 3 T (MRT; Intera, Philips Healthcare, Best, the Netherlands or High Field NMR Relaxometer, Stelar, Pavia, Italy) and 4.7 T (MRT; Bruker BioSpec, 70/20, Ettlingen, Germany) at site 1 and 7 T (MRT; Bruker BioSpec 70/30) at site 2. The T1 of bile was measured with all devices except at 4.7 T. The vendor‐specific optimized T1 relaxation measurement method was employed for each device.

For 1.41 and 3 T NMR devices, T1 was measured using standard software of the NMR with a two‐pulse IR sequence and a fixed relaxation delay of 5 x T1. The variable inversion times were automatically calculated based on an estimated T1 (T1e) value and ranged from 0.1 x T1e to 4 x T1e in eight steps for 1.41 T and from 0.01 x T1e to 4 x T1e in 16 steps for 3 T.

For 1.5 and 3 T MRI scanners, the SI of each set of rat samples were determined using the inversion recovery (IR) method. Regions of interest (ROIs) were placed manually in the first image of each sample and copied to the subsequent 11 inversion time images. To calculate T1, the average signal of each ROI was fitted to the inversion recovery signal evolution with dedicated in‐house software.^19^

For 4.7 and 7 T MRI scanners, SI were measured using a Rapid Acquisition with Relaxation Enhancement (RARE) saturation‐recovery fast‐spin echo sequence.^21^ ROIs were manually drawn on T1 maps generated from image data by ParaVision 6.0.1 software (Bruker Biospin) and ROI‐averaged values for T1 were taken.

T1 values obtained from all devices were converted to relaxation rates (R1s) (R1 = 1/T1). For liver tissue, the values of three samples per Mrp2‐KO animal were averaged for further data processing of the relaxivity.

### 2.3 Determination of gadolinium concentration in tissue and biofluids

At site 1, all the samples were subsequently weighed and mixed with 0.04 mL 1000 ppm yttrium (part no. 170368, Certipur ICP Standard, Merck KgaA, Darmstadt, Germany) as an internal standard. Then 1.2 mL of concentrated nitric acid (65% HNO_3_, Suprapur; Merck KgaA) and 0.8 mL of hydrogen peroxide (30% H_2_O_2_, Emsure; Merck KgaA) were added. Samples were digested by heating at 200°C and 800 W for 20 minutes in a microwave oven (MDS 2000; CEM, Kamp‐Lintfort, Germany). After cooling, 2 mL of deionized H_2_O was added. The gadolinium concentration was determined by inductively coupled plasma optical emission spectrometry (ICP‐OES; iCAP7600, Thermo‐Fischer Scientific GmbH, Dreieich, Germany). A calibration standard of 10 ppm gadolinium containing 10 ppm yttrium as an internal standard was utilized.

All samples taken at site 2 were weighed accurately and homogenized (1 g tissue/3 mL deionized water) using a Bullet Blender (Next Advance Model BB24). To the homogenized hepatocyte tissue, as well as to 0.025 mL of each blood, urine and bile sample, 0.07 mL of concentrated 65% nitric acid (TraceMetal Grade, Fisher Scientific, Waltham, MA, USA) and 0.03 mL of 30% hydrogen peroxide were added to each tube. Samples of hepatocytes, blood and bile were digested at 65°C and 1000 W for 30 minutes in a microwave. Urine samples were left at room temperature to digest overnight. All samples were centrifuged and then diluted to a final volume of 10 mL with 2% nitric acid before they were analyzed by inductively coupled plasma mass spectrometry (ICP‐MS; NexION 350X, Perkin Elmer, Waltham, MA, USA) coupled to a SC‐2 DX prepFAST auto sampler (Elemental Scientific, Omaha, NE, USA). Gadolinium ICP standard 1000 ppm (Agilent, part no. 5190‐8241) was used to construct calibration standards ranging from 1 to 200 ppb. Gd 158 isotope was used for further calculations. Tb 159 (Internal Standard Multi‐Element Mix 1, VHG Labs, Manchester, NH, USA) was applied as the ICP‐MS internal standard and supplied with continuous flow at a concentration of 20 ppb.

### 2.4 Relaxivity calculation

Relaxivity values were determined for blood collected 1 minute after gadoxetate injection, for urine and bile continuously collected from the time point of injection until animal sacrifice, and for liver collected 30 minutes p.i. Data from the 3 T MRI scanner and the 3 T NMR Analyzer were pooled. Relaxivity and the standard error were centrally calculated at site 1 for each field strength as the slope of a linear regression fit of the determined R1 versus measured gadolinium concentration with GraphPad Prism (GraphPad Prism 7, La Jolla, CA, USA).

### 2.5 Theoretical modeling of hepatocyte transporter function

An appropriate physiologically based pharmacokinetic (PBPK) model allows characterization of hepatic uptake and efflux kinetics of gadoxetate in vivo by input from quantitative DCE‐MRI data. To theoretically assess the potential impact of the r1 values in liver tissue and blood derived in this study on gadoxetate transport rate constant calculation, we considered a two‐compartmental high‐flow filtration model.^22^, ^23^

The model has an extracellular compartment “e” and a hepatocyte compartment “h” and assumes that “e” is in equilibrium with the body's blood pool. Transport rate constants for gadoxetate uptake from the extracellular compartment “e” into hepatocytes “h” and efflux into bile “b” are given as k_he_ and k_bh_, respectively, with v_e_ and v_h_ denoting compartmental extracellular volume fractions.

(1) $$\Delta R_{1}\left(t\right)=v_{e}\;\Delta R_{\text{1e}}\left(t\right)+\left(r_{\text{1h}}/r_{\text{1e}}\right)\;k_{\mathrm{he}}\;\exp\left(-t\;k_{\mathrm{bh}}/v_{h}\right)\;⊗\;\Delta R_{\text{1e}}\left(t\right)$$

The derivation of this equation is provided in the supporting information (Data S1).

Because v_e_ is treated as a known constant, the model is defined by two unknown parameters, A = (r_1h_/r_1e_) k_he_ and B = k_bh_/v_h_, which can be calculated by minimizing the difference between the left‐ and right‐hand sides of Equation 1. From A and B, the rate constants can then be determined:

(2) $$k_{\mathrm{he}}=A/\left(r_{\text{1h}}/r_{\text{1e}}\right);$$

(3) $$k_{\mathrm{bh}}=B\;v_{h}$$

## 3 RESULTS

### 3.1 Relaxation rate and gadolinium concentration

**FIGURE 1:**
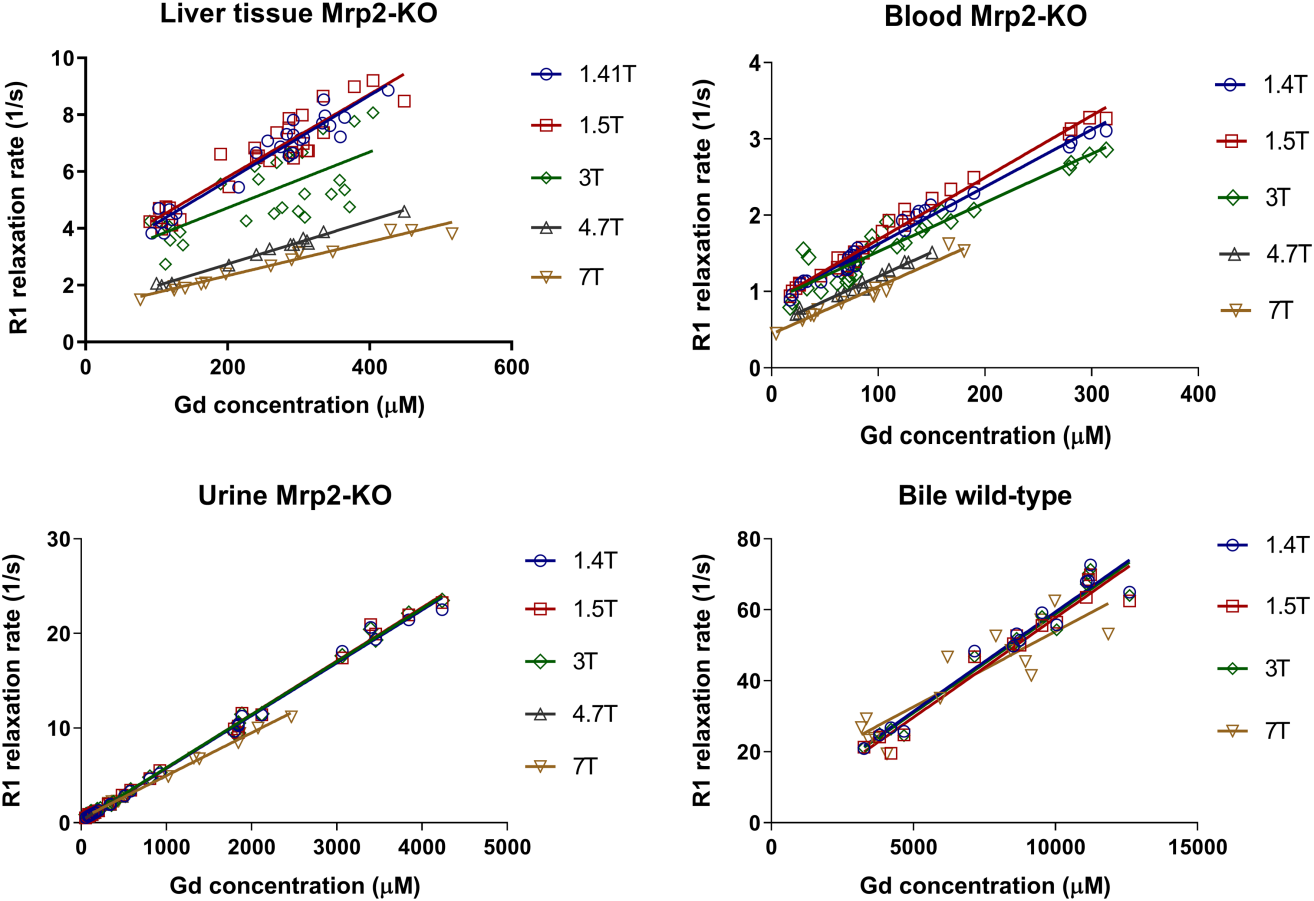
Plots of gadoxetate R1 versus gadolinium concentration of each rat in liver tissue (with gadoxetate depleted from blood and bile), blood and urine collected from Mrp2‐KO rats and in bile from wild‐type rats color‐coded for each field strength at 1.41, 1.5, 3.0, 4.7 and 7 T. The slope of a linear regression fit for relaxivity calculation is overlaid

The calculated R1 values plotted against the respective tissue or biofluid gadolinium concentrations at all magnetic field strengths for liver, blood at 1 minute p.i., urine (Mrp2‐KO rats) and for bile (wt rats) are shown in Figure 1 together with their linear regression fits for each field strength. Gadolinium concentrations in bile (Mrp2‐KO rats) ranged from 8 to 110 μM. These bile samples were not included in the relaxivity calculation due to low Gd concentrations.

For blood sampled at 30 minutes p.i., gadolinium concentrations for the lowest and highest administered gadoxetate doses ranged from 3.6 to 20.4 μM (Mrp2‐KO rats). Consequently, blood sampled at 30 minutes postgadoxetate injection was not included in the relaxivity calculation either, as the concentrations were not sufficiently high. On average, only 7.6% ± 4.4% of the gadolinium concentration measured at 1 minute p.i. was still present in blood at 30 minutes postgadoxetate injection. With regards to liver tissue, the 30 minute blood gadolinium concentration was on average 4.1% ± 1.5%.

**FIGURE 2:**
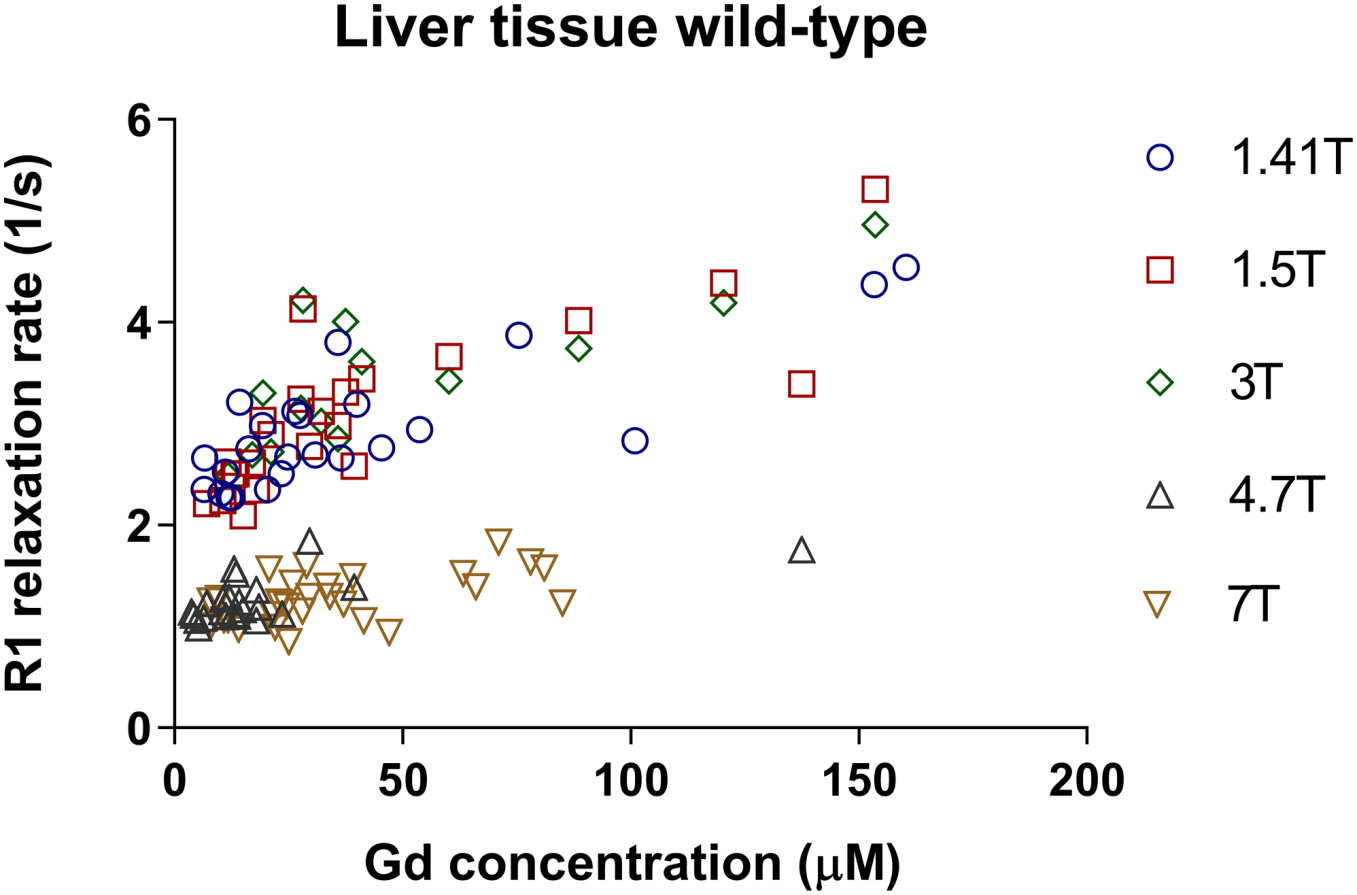
Plots of gadoxetate R1 versus gadolinium concentration of each rat in liver tissue collected from wild‐type rats color‐coded for each field strength at 1.41, 1.5, 3.0, 4.7 and 7 T

In Figure 2, the calculated R1 values plotted against the respective tissue gadolinium concentrations are presented for the liver tissue of wt rats. The coefficients of determination (R^2^) of the goodness of fit were in a range from 0.65 to 0.29 across all studied field strengths.

### 3.2 Relaxivity results

**FIGURE 3:**
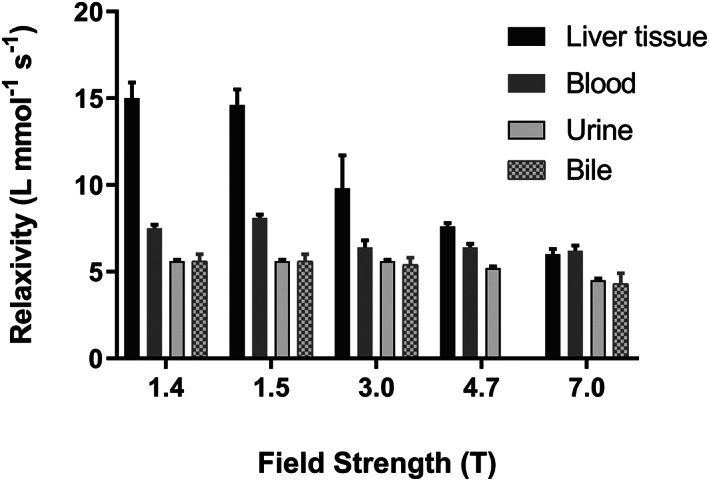
Gadoxetate r1 in liver tissue (with gadoxetate depleted from blood and bile), blood and urine collected at both sites from Mrp2‐KO rats and bile from wild‐type rats determined at 1.41, 1.5, 3.0, 4.7 (except for bile) and 7 T. Error bars show + standard error

Average gadoxetate r1 values derived from linear regressions of R1 values versus gadolinium concentrations at the magnetic field strengths of 1.41, 1.5, 3, 4.7 and 7 T are shown in Table 2 and visualized in Figure 3, each with their respective standard error of the mean.

**TABLE 2:** Gadoxetate r1 in liver tissue (with gadoxetate depleted from blood and bile), blood and urine collected from Mrp2‐KO rats and in bile from wild‐type rats determined at 1.41, 1.5, 3.0, 4.7 (except for bile) and 7 T. Shown are the mean +/− standard error of the fit slope

Relaxivity (L mmol^−1^ s^‐1^)	1.41 T	1.5 T	3 T	4.7 T	7 T
**Liver tissue**	15.0 ± 0.9	14.6 ± 1.1	9.8 ± 1.9	7.6 ± 0.2	6.0 ± 0.3
**Blood**	7.5 ± 0.2	8.1 ± 0.2	6.4 ± 0.4	6.4 ± 0.2	6.2 ± 0.3
**Urine**	5.6 ± 0.1	5.6 ± 0.1	5.6 ± 0.1	5.2 ± 0.1	4.5 ± 0.1
**Bile**	5.6 ± 0.4	5.6 ± 0.4	5.6 ± 0.4	n.d.	4.3 ± 0.6

Abbreviations: n.d, not determined.

The coefficients of determination (R^2^) of the goodness of fit in liver tissue were 0.93 at 1.41 T, 0.88 at 1.5 T, 0.51 at 3 T, 0.99 at 4.7 T and 0.96 at 7 T. For 1 minute blood, urine and bile, R^2^ was on average 0.96 ± 0.03, 1.00 ± 0.001 and 0.91 ± 0.08, respectively, across all studied field strengths.

The r1 of gadoxetate in liver tissue was found to be highest at 1.41 and 1.5 T (15.0 ± 0.9 L mmol^−1^ s^−1^ at 1.41 T; 14.6 ± 1.1 L mmol^−1^ s^−1^ at 1.5 T) and decreased with increasing field strength to 6.0 ± 0.3 L mmol^−1^ s^−1^ at 7 T. The gadoxetate r1 values in blood were lower than in liver tissue and were less influenced by field strength (7.5 ± 0.2 L mmol^−1^ s^−1^ at 1.41 T, decreasing slightly to 6.2 ± 0.3 L mmol^−1^ s^−1^ at 7 T). The lowest values for gadoxetate r1 were found for urine and bile. The gadoxetate relaxivities of both fluids decreased by 20% for urine and 23% for bile with an increase in the magnetic field from 1.41 to 7 T. The derived relaxivities were obtained with a relative standard error of 3.9% in blood, ±1.9% in urine, ±8.7% in bile and within ±8.1% in liver tissue, averaged over all measured field strengths.

### 3.3 Gadoxetate liver transport rate constants

As shown by Equation 2, the uptake rate k_he_ of gadoxetate from blood into hepatocytes directly scales with the ratio of the relaxivity of hepatocytes to blood (r_1h_/r_1e_).^24^, ^25^ Taking the determined r1 in liver as the r1 of hepatocytes (as discussed below), the ratio of gadoxetate r1 in hepatocytes to blood (r_1h_/r_1e_) is 2 at 1.41 T and 1.8 at 1.5 T, gradually decreasing with increasing magnetic field strength, being 1.5 at 3 T, 1.2 at 4.7 T and 0.97 at 7 T.

Note that the biliary excretion rate k_bh_ is not affected by the different relaxivities and therefore does not require correction (Equation 3).

## 4 DISCUSSION

In this study, we provide experimentally determined ex vivo gadoxetate r1 relaxivity values for rat liver tissue, which should approximate hepatocytes, and for blood, urine and bile across a range of magnetic field strengths from 1.41 to 7 T, relevant for both preclinical and clinical MRI scanners. We found a 2‐fold higher r1 for gadoxetate in liver tissue compared with blood at 1.41 T. We determined a strong field strength dependency for gadoxetate r1 in rat liver tissue, resulting in a decreasing ratio of r1 in liver to r1 in blood with increasing field strength, reaching ~1 at 7 T. Furthermore, to demonstrate the relevance of our findings on the MRI‐based determination of gadoxetate transport rate constants, we used a kinetic model for the calculation of MRI‐derived hepatic uptake and excretion rate constants. We showed a direct scaling of uptake rate constants with the ratio of gadoxetate r1 in hepatocytes to blood.

Liver tissue comprises hepatocytes, blood, bile and small contributions from other liver‐resident, nonparenchymal cells. To derive gadoxetate r1 from hepatocytes only, rather than from all compartments in the entire liver tissue, we chose to utilize the Mrp2 knockout rat strain SD‐*Abcc2* ^*tm1sage*^.^26^, ^27^, ^28^ This rat strain bears a loss of function mutation in the Mrp2 transporter, which in wt animals eliminates gadoxetate from hepatocytes into bile.^29^ Due to the loss of function mutation, gadoxetate excretion from hepatocytes into bile will be impaired, thereby avoiding gadoxetate from bile contributing to the overall signal in the liver. The second main compartment, with potentially high gadoxetate concentrations outside hepatocytes, is blood. To eliminate this constraint, we considered 30 minutes between gadoxetate injection and animal sacrifice as sufficient to reduce the gadoxetate blood level in the liver to minimal values, as the elimination half‐life of gadoxetate in wt rat blood is very short (10.2 ± 1.9 minutes).^30^ Our study shows that the remaining gadoxetate concentration in blood reached 4.1% ± 1.5% of the values determined in the liver. If we also neglect contributions from other cells resident in the liver,^24^ as those are not known to take up gadoxetate,^25^ and make only a minor contribution to the overall liver volume, the T1 values measured from liver samples taken in our experiments emanate mostly from hepatocytes. Derived r1 values of the measured liver tissue are therefore representative of gadoxetate r1 in hepatocytes.

The r1 results we obtained differ between the studied tissue compartments and show distinct dependencies on magnetic field strength. These differences are mainly induced by the environmental conditions surrounding a GBCA and disparate protein binding of gadoxetate, which is known to affect the rotational correlation time of GBCAs and thus their relaxivity.^19^, ^31^, ^32^ Hepatocytes contain higher protein levels than blood or the rather aqueous bile and urine, and also contain a protein composition specific to the intracellular compartment.^33^, ^34^ Potential higher protein binding would lead to the observed increased relaxivity towards lower field strengths. This is supported by data obtained from other GBCAs with known protein‐binding properties (eg, gadofosveset) that exhibit a highly field strength‐dependent r1.^19^, ^20^ Converse to other studies^35^, ^36^ we did not observe any quenching effect of the longitudinal relaxivity caused by the internalization of gadoxetate into hepatocytes, which may take place at high GBCA concentration entrapped in small compartments like endosomes. In such cases the relationship between relaxation rate and the Gd concentration determined would not be linear. Viscosity is another widely accepted property affecting the rotational correlation time, and thus relaxivity. It has been shown that increasing the amounts of macromolecules, where there is no specific interaction between the macromolecule and the metal complex, impacts on (micro)viscosity and thus leads to higher relaxivity.^37^, ^38^ A trend for r1 over a field strength range that is relevant to MR imaging in animals and humans, and which is similar to known protein‐binding GBCAs, becomes apparent. Shuter et al^18^ reported a gadoxetate r1 for the liver tissue of wt rats of 10.7 ± 0.5 kg mmol^−1^ s^−1^ at 1.5 T, which is lower when compared with values found in our study for hepatocytes. This could be attributed to our experimental design, which was specifically created to exclude gadoxetate from all liver compartments except hepatocytes. Because this approach was not followed by Shuter et al,^18^ they provide a gadoxetate r1 for overall liver tissue, averaged over all tissue compartments.

Our experimental data for liver tissue from wt rats highlight the importance of eliminating gadoxetate from other liver compartments than hepatocytes when aiming to determine gadoxetate r1 in this tissue compartment. The plot of the calculated relaxation rates against gadolinium concentration (Figure 2) shows a high data variability in wt animals. This was most probably caused by contamination of liver samples with bile containing different gadoxetate concentrations at a rather high level. In addition, bile duct cannulation, which is required to determine the gadoxetate concentration in bile, potentially leads to partially impaired bile flow and also influences gadoxetate excretion from hepatocytes to bile to a variable extent in individual animals. So the ratio of gadoxetate concentration in hepatocytes versus bile may significantly differ between individual animals, introducing a further source of variability to relaxivity determination.

In blood, the reduction of r1 towards higher field strength is known to be caused by gadoxetate's plasma protein binding. Bile and urine are basically protein‐free fluids. Therefore, gadoxetate remains unbound and exhibits the lowest r1 of all the studied compartments, identical to its r1 in water. In overall accordance with the literature, r1 in bile and urine exhibits only slight field strength dependence.^19^ Minor deviations observed between r1 values of various GBCAs reported from studies are probably caused by different measurement systems and instrumentation.

As shown in Equation 1, the ratio of gadoxetate r1 in hepatocytes to its r1 in blood impacts measured uptake rate constants into hepatocytes.^22^, ^23^ This, or equivalent kinetic models,^39^, ^40^, ^41^ require knowledge of gadoxetate concentrations in biological compartments, which can be derived from MRI signal intensities with known signal evolution equations and r1. The accuracy of gadoxetate concentration determination in vivo by MRI depends not only on utilizing the correct relaxivity, but also on the quality of the measurement itself (eg, accurate B1). In recent reports, kinetic models were applied assuming gadoxetate r1 to be identical in blood and in hepatocytes.^14^, ^39^ Our results show that this leads to an overestimation of uptake rate constants at the respective magnetic field strengths. The overestimation diminishes with higher field strength from a factor of 2 at 1.41 T to a factor of 1 at 7 T. Consequently, numerical values at different field strengths are not comparable, unless properly accounting for relaxivity difference. This has significant implications for translational studies where preclinical measurements at high field are used to make predictions for measurements at a lower clinical field strength. Fortunately, since the effect is merely a scaling factor, results from previous studies can easily be corrected by applying a respective field‐dependent correction factor.

The numerical values provided here for gadoxetate r1 in hepatocytes are appropriate with which to estimate gadoxetate concentrations in liver tissue and are applicable for transport rate constant determination in animal liver models and patients. In functionally impaired liver tissue, gadoxetate hepatocyte transport activity may be altered, which is reflected in liver tissue enhancement.^40^ Decrease in signal enhancement may be caused by attenuated gadoxetate uptake by hepatocytes or a lower number of normal hepatocytes, while impairment of gadoxetate excretion from hepatocytes may result in prolongation of liver enhancement. The latter condition may lead to a very high gadoxetate concentration inside hepatocytes and is represented in our experiments by using Mrp2‐KO rats, since their depletion of Mrp2 transporter displays compromised biliary excretion of gadoxetate. The linear correlation between relaxation rate and Gd concentration, which we observed despite very high gadoxetate concentrations in Mrp2‐KO liver tissue, shows that the derived gadoxetate relaxivity in our model is not influenced by quenching or other effects, which would lead to a nonlinear relation of relaxation rate and Gd concentration.

It is well known that the total protein level and its composition changes in a pathological state.^42^, ^43^ in vitro studies^44^ at clinical field strengths allude to a strong response of a GBCA's relaxivity with pathological deviations from normal plasma‐protein concentrations in aqueous solutions; however, translation to our ex vivo experiments and the effect on in vivo imaging cannot be determined. In our study, we did not measure protein levels, or reflect pathological conditions with known severe changes of the latter. An entire prediction of a protein level‐dependent relaxivity response is beyond the scope of this study. Nonetheless, our relaxivity data can serve as a benchmark for further investigations reflecting various disease constraints.

Some limitations to our study should be noted. First, we chose 30 minutes as the time point for the determination of r1 in liver tissue. Approximately 4% of total gadoxetate concentration in our liver tissue samples remained in blood. The contribution of gadoxetate in blood on liver tissue r1 was therefore shown to be low; however, a minor impact on relaxivity in hepatocytes cannot be fully excluded. Second, Mrp2‐KO rats instead of wt rats were employed for determination of gadoxetate relaxivity in hepatocytes, as we aimed to avoid gadoxetate contamination from bile. It has to be considered that this loss of function mutation may lead to a slightly altered protein composition inside hepatocytes, potentially leading to a modest variation in gadoxetate r1 values from, for example, Wistar rats, which are commonly used in biomedical research.

In conclusion, we present here numerical values for gadoxetate r1 in tissue and biofluids and show that the ratio of gadoxetate r1 in hepatocytes to its r1 in blood is dependent on field strength. Moreover, we derived correction factors for rate constants at all relevant magnetic field strengths. Our findings should be considered for quantitative gadoxetate‐enhanced liver MRI and also have an impact upon kinetic modeling‐based determination of gadoxetate hepatic uptake rate constants, which is of relevance when comparing respective data from measurements at different magnetic field strengths.

## FUNDING INFORMATION

Funds were received from the Innovative Medicines Initiatives 2 Joint Undertaking under grant agreement No 116106 (IB4SD‐TRISTAN). This Joint Undertaking receives support from the European Union's Horizon 2020 Research and Innovation Programme and EFPIA.

## Supporting information


**Data S1** Supporting information
