# Supplementary material for: Ex vivo gadoxetate relaxivities in rat liver tissue and blood at five magnetic field strengths from 1.41 to 7 T
Source: NMR Biomed. 2020 Aug 26;34(1):e4401. doi: 10.1002/nbm.4401 (PMC7757196; doi:10.1002/nbm.4401)
Supplement: Supplementary file 1 — Data S1 Supporting information [file NBM-34-e4401-s001.docx]

Supplementary Material A

Derivation of the kinetic model

The concentration c_e_(t) in “e” can be measured in a large vessel or a reference tissue. The concentration c_h_(t) in “h” can then be determined by expressing mass conservation for the contrast agent:

[Eq. S1] v_h_ dc_h_/dt = k_he_ c_e_ – k_bh_ c_h_

Here v_h_ is the volume fraction of “h” and k_he_ and k_bh_ are the rate constants for intracellular uptake from “e” to “h”, and for excretion from “h” to bile, respectively. Since c_e_(t) is known, Eq. [1] can be solved directly using the convolution product ⊗:

[Eq. S2] v_h_ c_h_(t) = k_he_ exp(-t k_bh_/v_h_) ⊗ c_e_(t)

Assuming the water exchange between the liver tissue compartments is fast, the relaxation rate R1 of liver is a weighted average of the compartments “e”, “h” and any other tissue components “x” that are not accessible to the contrast agent:

[Eq. S3] R_1_ = v_e_R_1e_ + v_h_R_1h_ + v_x_R_1x_

In the presence of a contrast agent, the relaxation rates of “e” and “h” increase linearly with concentration following R_1e_ = R_10,e_ + r_1e_c_e_ and R_1h_ = R_10,h_ + r_1h_c_h_. Inserting this in Eq. 3 and using that pre-contrast R_10_ = v_e_R_10,e_ + v_h_R_10,h_ + v_x_R_1x_ we find an expression for ΔR_1_ = R_1_ - R_10_ as a function of the concentrations in the compartments:

[Eq. S4] ΔR_1_ = r_1e_ v_e_c_e_ + r_1h_ v_h_c_h_

Since c = v_e_c_e_ + v_h_c_h_, Eq. [4] reduces to the well-known relation ΔR_1_ = r_1_c in the particular case that r_1e_ = r_1h_ = r_1_. Hence, Eq. 4 expresses a more general relationship that also holds true for tissues with compartments that have different relaxivities. Note that in this case the tissue concentration c can no longer be derived directly from ΔR_1_ because the change in relaxation rate also depends on how c is distributed over the individual compartments.

Inserting now Eq. [2] into Eq. [4], and using that c_e_ is measured as ΔR_1e_ / r_1e_ from a reference region in blood, we find the final result:

[Eq. S5] ΔR_1_(t) = v_e_ ΔR_1e_(t) + (r_1h_/r_1e_) k_he_ exp(-t k_bh_/v_h_) ⊗ ΔR_1e_(t)
